# Accessability to mental health services during pandemic period

**DOI:** 10.1192/j.eurpsy.2023.413

**Published:** 2023-07-19

**Authors:** A. Mihai, H. Gungor

**Affiliations:** ^1^Psychiatry, George Emil Palade University of Medicine, Pharmacy, Science and Technology; ^2^Psychiatry, Psychiatric Clinic I, County Emergency Hospital Mures, Targu Mures, Romania

## Abstract

**Introduction:**

A try to protect people diagnosed with dementia, from the COVID virus, during pandemic period could easily lead to isolation and hinder them to reach the needed medical care.

**Objectives:**

Research question refer to accessibility during pandemic to psychiatric services of patients with dementia.

**Methods:**

The consultations of patients with dementia of an outpatient setting where analyzed before (2019-2020) and during pandemic (2020-2021). We evaluate the consultations frequency, characteristics of patients, comorbidities and treatment changes.

**Results:**

A total number of 965 consultation (493/472) were analyzed. There was no statistically significant difference between the number of consultations before and during the pandemic, which means that the patients had access to medical assistance with the same frequency as before the pandemic. (p=0.63) There was no statistically significant difference in different environment groups (urban/rural). (p= 0.53) Telemedicine (videoconference) and phone consultation where also used during pandemic period. 63,6% of those in rural area chosed telemedicine and 9% phone consultation. Significantly more patients from the urban area were consulted on-site during the Covid pandemic. (p=0.04) Despite the risk, patients with comorbidities had visited more often the hospital during the COVID-19 pandemic. (p=0.012) In 39 % of cases there was worsen evolution and a drug change: most frequent add it a hypnotic or an antipsychotic.

**Conclusions:**

The accessibility of the psychiatry clinic during the pandemic was at all times possible for dementia patients. Telemedicine ensured an ongoing consultation flow for the patients.

**Disclosure of Interest:**

None Declared

